# COVID-19 and Cognitive Decline in Older Adults with High-Cardiovascular Risk: A Post Hoc Analysis

**DOI:** 10.14336/AD.2024.0380

**Published:** 2025-05-21

**Authors:** Sangeetha Shyam, Carlos Gómez-Martínez, Jiaqi Ni, José J. Gaforio, Miguel Ángel Martínez-González, Dolores Corella, J. Alfredo Martínez, Ángel M. Alonso-Gómez, Julia Wärnberg, Jesús Vioque, Dora Romaguera, José López-Miranda, Ramon Estruch, Francisco J Tinahones, José Lapetra, Lluís Serra-Majem, Aurora Bueno-Cavanillas, Josep A. Tur, Vicente Martín-Sánchez, Xavier Pintó, Miguel Delgado-Rodríguez, Pilar Matía-Martín, Josep Vidal, Clotilde Vázquez, Lidia Daimiel, Emilio Ros, Fernando Fernandez-Aranda, Adrián Hernández-Cacho, Pilar Buil-Cosiales, Jose V Sorli, Olga Castañer, Antonio Garcia-Rios, Alejandro Oncina-Canovas, Napoleón Pérez-Farinós, Mar Nafria, Rosa Casas, Silvia Martínez-Diz, Lucas Tojal-Sierra, Gómez-Pérez AM, Estefania Toledo, Rebeca Fernández-Carrión, Álvaro Morán Bayón, Jose David Torres-Peña, Laura Compañ-Gabucio, Zenaida Vázquez-Ruiz, Nancy Babio, Montserrat Fitó, Jordi Salas-Salvadó

**Affiliations:** ^1^Universitat Rovira i Virgili, Departament de Bioquímica i Biotecnologia, Unitat de Nutrició, Reus, Spain.; ^2^Institut d'Investigació Sanitària Pere Virgili (IISPV), Reus, Spain.; ^3^Centro de Investigación Biomédica en Red Fisiopatología de la Obesidad y la Nutrición (CIBEROBN), Institute of Health Carlos III, Madrid, Spain.; ^4^CIBER de Epidemiología y Salud Pública (CIBERESP), Instituto de Salud Carlos III, Madrid, Spain.; ^5^Departamento de Ciencias de la Salud, Instituto Universitario de Investigación en Olivar y Aceites de Oliva, Universidad de Jaén, Jaén, Spain.; ^6^University of Navarra, Department of Preventive Medicine and Public Health, IdiSNA, Pamplona, Spain.; ^7^Department of Nutrition, Harvard T.H. Chan School of Public Health, Boston, MA, USA.; ^8^Department of Preventive Medicine, University of Valencia, Valencia, Spain.; ^9^Department of Nutrition, Food Sciences, and Physiology, Center for Nutrition Research, University of Navarra, Pamplona, Spain.; ^10^Precision Nutrition and Cardiometabolic Health Program. IMDEA Food, CEI UAM + CSIC, Madrid, Spain.; ^11^Bioaraba Health Research Institute, Cardiovascular, Respiratory and Metabolic Area; Osakidetza Basque Health Service, Araba University Hospital University of the Basque Country UPV/EHU, Vitoria-Gasteiz, Spain.; ^12^EpiPHAAN research group, University of Málaga - Instituto de Investigación Biomédica en Málaga (IBIMA), Málaga, Spain.; ^13^Instituto de Investigación Sanitaria y Biomédica de Alicante. Universidad Miguel Hernández (ISABIAL-UMH). Alicante, Spain.; ^14^Health Research Institute of the Balearic Islands (IdISBa), Palma de Mallorca, Spain.; ^15^Lipids and Atherosclerosis Unit, Department of Internal Medicine, Maimonides Biomedical Research Institute of Cordoba (IMIBIC), Reina Sofia University Hospital, University of Cordoba, Av. Menéndez Pidal s/n, 14004 Cordoba, Spain.; ^16^Department of Internal Medicine, Institut d’Investigacions Biomèdiques August Pi Sunyer (IDIBAPS), Hospital Clinic, and Institut de Recerca en Nutrició i Seguretat Alimentaria (INSA-UB); University of Barcelona, Barcelona, Spain.; ^17^Virgen de la Victoria Hospital, Department of Endocrinology, Instituto de Investigación Biomédica de Málaga (IBIMA). University of Málaga, Málaga, Spain.; ^18^Department of Family Medicine, Research Unit, Distrito Sanitario Atención Primaria Sevilla, Sevilla, Spain.; ^19^Research Institute of Biomedical and Health Sciences (IUIBS), University of Las Palmas de Gran Canaria & Centro Hospitalario Universitario Insular Materno Infantil (CHUIMI), Canarian Health Service, Las Palmas de Gran Canaria, Spain.; ^20^Department of Preventive Medicine and Public Health, University of Granada, Granada, Spain.; ^21^Instituto de Investigación Biosanitaria ibs. GRANADA, Granada, Spain.; ^22^Research Group on Community Nutrition & Oxidative Stress, University of Balearic Islands, Research Institute of Health Sciences (IUNICS), Palma de Mallorca, Spain.; ^23^Institute of Biomedicine (IBIOMED), University of León, León, Spain.; ^24^Lipids and Vascular Risk Unit, Internal Medicine, Hospital Universitario de Bellvitge-Institut d'Investigacions biomèdiques de Bellvitge (IDIBELL), Hospitalet de Llobregat, Barcelona Spain.; ^26^Department of Endocrinology and Nutrition, Instituto de Investigación Sanitaria Hospital Clínico San Carlos (IdISSC), Madrid, Spain.; ^27^CIBER Diabetes y Enfermedades Metabólicas (CIBERDEM), Instituto de Salud Carlos III (ISCIII), Madrid, Spain.; ^28^Department of Endocrinology, Institut d` Investigacions Biomédiques August Pi Sunyer (IDIBAPS), Hospital Clinic, University of Barcelona, Barcelona, Spain.; ^29^Department of Endocrinology and Nutrition, Hospital Fundación Jimenez Díaz. Instituto de Investigaciones Biomédicas IISFJD. University Autonoma, Madrid, Spain.; ^30^Nutritional Control of the Epigenome Group. Precision Nutrition and Obesity Program. IMDEA Food, CEI UAM + CSIC, Madrid, Spain.; ^31^Departamento de Ciencias Farmacéuticas y de la Salud, Faculty de Farmacia, Universidad San Pablo-CEU, CEU Universities, Boadilla del Monte, Spain.; ^32^Lipid Clinic, Department of Endocrinology and Nutrition, Institut d’Investigacions Biomèdiques August Pi Sunyer (IDIBAPS), Hospital Clínic, Barcelona, Spain.; ^33^Psychology Department, University Hospital of Bellvitge-ICS, Hospitalet del Llobregat (Barcelona), Spain.; ^34^Servicio Navarro de salud, Primary Health Care, Navarra, Spain.; ^35^Unit of Cardiovascular Risk and Nutrition, Institut Hospital del Mar de Investigaciones Médicas Municipal d’Investigació Médica (IMIM), Barcelona, Spain.; ^36^Preventive Medicine and Public Health Service, Hospital Universitario Virgen de las Nieves, Granada, Spain.;

**Keywords:** COVID-19, Cognition, Older adults, PREDIMED-Plus

## Abstract

Cognitive decline has been reported as a short-term sequela in patients hospitalized for coronavirus disease-19 (COVID-19). Whether COVID-19 is associated with late cognitive impairment in older free-living individuals with high cardiovascular risk, a group at greater risk of cognitive decline, is unknown. We determined this association of COVID-19 through a longitudinal evaluation of post-COVID-19 cognitive performance and impairment as post hoc analysis in 5,179 older adults (48% female) with mean (SD) age 68.5 (5.0) years, body mass index 31.7 (3.7) kg/m^2^, harboring ≥ 3 criteria for metabolic syndrome (e.g., hypertension, hyperlipidemia, hyperglycemia etc.) enrolled in PREDIMED-Plus trial. Pre- and post-COVID-19 cognitive performance was ascertained from scheduled assessments conducted using a battery of neuropsychological tests, including 5 domains: Global Cognitive Function, General Cognitive Function, Execution Function, Verbal Fluency and Attention domains, which were standardized for the cohort. Cognitive impairment was defined as the bottom 10 percentile of the sample. Multivariable linear and logistic regression models assessed the association of COVID-19 with cognitive decline and impairment, respectively. After a mean 50-week follow-up, no significant associations were observed between COVID-19 status and post-COVID-19 scores of all tapped neuropsychological domains, except Global Cognitive Function (GCF). When fully adjusted, COVID-19 was marginally associated with higher (better) post-pandemic GCF score (βadj (95% CI): 0.06 (0.00, 0.13) p=.05). However, the odds for post-COVID-19 cognitive impairment in GCF domain were not associated with the disease (ORadj (95% CI): 0.90 (0.53, 1.51) p=.68). In the PREDIMED-Plus cohort, COVID-19 status and cognitive impairment determined 50 weeks post-infection showed no association in older adults at high cardiovascular risk. This suggests that cognitive changes observed shortly after COVID-19 revert over time. However, cautious interpretation is warranted as these data were obtained within the framework of a clinical trial encouraging a healthy lifestyle.

## INTRODUCTION

Cognitive impairment, a major determinant of poor health and mortality in older adults [1], is a common neuropsychological sequela of coronavirus disease 2019 (COVID-19)[2-5]. Cognitive deficits post-COVID-19 have been reported in several cognitive domains [6], associating COVID-19 with cognitive impairment and dementia [6, 7]. These COVID-19-related cognitive deficits occur through structural and functional brain changes [2, 3].

Several individual-level characteristics including cardiovascular risks such as obesity, diabetes, hypertension, and depression are thought to influence COVID-19-related cognitive decline [8]. As older age is associated with an increased risk for severe COVID-19 [9], infection-related cognitive deficits could be a health concern among older adults, specifically those with cardiovascular risk factors. Hence, we examined older adults enrolled in the PREDIMED-Plus trial, the association of COVID-19 status with cognitive impairment measured at 1-year post-infection. From a public health perspective, such a long-term assessment in older adults with cardiometabolic risks informs if there is a need for sustained cognitive support in older adults with a high propensity for dementia.

## MATERIALS AND METHODS

### Study design and participants

This longitudinal analysis was conducted within the framework of PREDIMED-Plus [10], an ongoing, multicenter, randomized controlled lifestyle trial in Spain for the primary prevention of cardiovascular disease. Eligible participants were older adults (55-75 years) with overweight/obesity (included BMI range: 27-40 kg/m^2^) and ≥3 criteria for metabolic syndrome (MetS).

The trial recruited participants between 2013 and 2016, who were randomized to an intervention (IG) or control group (CG). IG encouraged energy-reduced Mediterranean diet (MedDiet) and physical activity and provided behavioural support to achieve/maintain weight loss. CG followed an *ad libitum* MedDiet. Participants provided written informed consent to a protocol approved by the institutional review boards of the participating centers (www.predimedplus.com).

### Exposure: COVID-19 status

The occurrence of COVID-19 was defined from the pandemic onset to 31^st^ December 2021 as adjudicated by the PREDIMED-Plus Clinical Event Ascertainment Committee. The ascertainment was based on the annual review of participant medical records by collaborating physicians [11], in accordance with CDC 2020 guidelines using PCR SARS-Cov-2, IgG tests [12]. Participants who did not have a confirmed COVID-19-positive diagnosis were considered COVID-19-negative. Subsequently, COVID-19 status was established dichotomously (positive/negative) and defined as exposure.

### Identifying pre- and post-COVID-19 data

For COVID-19-positive participants, the last available data prior to COVID-19 diagnosis was the pre-COVID-19 assessment, and the first evaluation after the COVID-19 diagnosis date was the post-COVID-19 assessment. In COVID-19 negative participants, the most recent data before the first known COVID-19 case in Spain (31^st^ January 2020) was the pre-COVID-19, and the subsequent available measurement was the post-COVID-19 data. The duration between pre- and post-COVID-19 cognitive assessment visits was calculated in weeks.

### Outcome Ascertainment

#### Cognitive function assessment and post-COVID-19 cognitive impairment

In PREDIMED-Plus, a neuropsychological battery of 8 cognitive tests assessed cognitive function at baseline and every 2 years [10]. These cognitive tests psychometrically evaluated for Spanish populations include the Mini-Mental State Examination (MMSE)[13], the Clock Drawing Test (CDT) [14], the Verbal Fluency Test animals (VFT-a) and letter “p” (VFT-p) categories [15], the Digit Span Test forward (DST-f) and backward (DST-b) sections, and the Trail Making Test A (TMT-A) and B (TMT-B) sections [16].

From these 8 tests, 5 cognitive domains were derived using z-scores, as prescribed in neuropsychological handbooks [17, 18]. These included the Global Cognitive Function (GCF), General Cognitive Function (genCF), Executive Function, Verbal Fluency, and Attention domains. Global Cognitive Function is defined as a composite of all 8 tests, with inversion of TMT-A and TMT-B scores, as higher scores reflect lower cognitive performance in these two tests. The General Cognitive Function domain included the MMSE and the CDT, the Executive Function domain included the VFT-a, VFT-p, DST-b, and TMT-B, the Verbal Fluency domain included the VFT-a and VFT-p, and the Attention domain included the DST-f and TMT-A tests.

#### Post-COVID-19 cognitive assessments.

Post-COVID-19 cognitive function was evaluated as aggregate performance in 5 cognitive domains assessed at the post-COVID-19 visit and estimated as z-score (number of SDs from the cohort mean, at pre-COVID-19 visit).

Global Cognitive Function composite score at the post-COVID-19 assessment was obtained using the following formula: Global Cognitive Function (post-COVID-19) = [zMMSE post-COVID-19 + zCDT post-COVID-19 + zVFT-a post-COVID-19 + zVFT-p post-COVID-19 + zDST-f post-COVID-19 + zDST-b post-COVID-19 + (-zTMT-A post-COVID-19) + (-zTMT-B post-COVID-19)]/8. Thereafter, the Global Cognitive Function composite score at the post-COVID-19 assessment was further standardized using the mean and standard deviation of the Global Cognitive Function composite score at the pre-COVID-19 assessment [19].

Post-COVID-19 cognitive impairment for each cognitive domain was determined as a dichotomous variable (yes/no) using the bottom 10th percentile of cognitive function [20].

**Table 1:** Participant characteristics at the pre-COVID-19 visit according to COVID-19 status.

Characteristics	COVID Status			
	Total population (n=5,179)	Negative (n=4,731)	Positive (n=448)	p-value
**Sex (female)** ^b^	2,473 (47.8%)	2,291 (48.4%)	182 (40.6%)	<0.01
**Age (in years)** #	68.5 ± 5.0	68. 4 ± 5.0	69.1 ± 5.1	0.01
**Allocation to Intervention arm** ^b^	2,488 (48.04%)	2,272 (48.0%)	216 (48.2%)	0.94
**Educational level** ^b^				0.04
**Primary school or less**	2,570 (49.62%)	2,373 (50.2%)	197 (44. 0%)	
**High school**	1,498 (28.92%)	1,356 (28.7%)	142 (31.7%)	
**College**	1,111 (21.45%)	1,002 (20.75%)	109 (24.3%)	
**Marital status** #				0.54
**Single, divorced or separated**	633 (12.2%)	583 (12.3%)	50 (11.2%)	
**Married**	3,959 (76.4%)	3, 607 (76.2%)	352 (78.6%)	
**Widowed**	587 (11.3%)	541 (11. 4%)	46 (10.3%)	
**Smoking status** ^b^				0.001
**Never smoker**	2,331 (45.0%)	2,155 (45.6%)	176 (39.3%)	
**Former smoker**	2,237 (43.19%)	2,007 (42.4%)	230 (51.3%)	
**Current smoker**	611 (11.80%)	569 (12.0%)	42 (9.4%)	
**Physical activity (METs min/week)** #	3,063 ± 2,450	3,043 ± 2,424	3,281 ± 2,698	0.05
**Alcohol intake (g/day)** #	9.6 ± 13.3	9.5 ± 13.3	10.3 ± 13.9	0.22
**MeDiet adherence score (er-MEDAS; range: 0-17 points)** #	11.7 ± 2.8	11.6 ± 2.8	11.8 ± 2.6	0.21
**Body mass index (kg/m^2^)** #	31.7 ± 3.7	31.7 ± 3.7	32.1 ± 3.7	0.04
**Hypertension** ^b^	4,332 (83.65%)	3,968 (83. 9%)	364 (81.3%)	0.15
**Hyperlipidemia** ^b^	3,619 (69.88 %)	3,317 (70.1%)	302 (67.4%)	0.23
**Type 2 diabetes** ^b^	1,559 (30.1%)	1,409 (29.8%)	150 (33. 5%)	0.10
**Depressive symptomatology** #	715 (13.82%)	664 (14.0%)	51 (11.4%)	0.12
**Received 1 dose of COVID-19**# **vaccine before event**	39 (0.75%)	0 (0%)	39 (8.7%)	<0.001
**Time from COVID-19 status ascertainment to post-COVID-19 cognitive assessment (weeks)**	43.7 ± 28.6	43.1 ± 28.5	49.9 ± 29.4	<0.001
**Time between pre- and post-COVID-19 cognitive assessments (weeks)** *	105.5 ± 6.2	105.5 ± 6.2	104.2 ± 6.1	<0.001

Abbreviations: MeDiet, Mediterranean diet; er-MEDAS, 17-point energy-restricted Mediterranean Diet Adherence Screener score. Data are n (%) or mean ± SD for categorical and quantitative variables, respectively. Chi-square is used for categorical variables and t-test for quantitative variables.

* The time was estimated for Global Cognitive Function domain measurements (n=4,838).

b Data obtained at baseline

# Measured at the pre-COVID-19 visit

*Covariates*

Sociodemographic, lifestyle, and history of disease covariates data were assessed by trained staff and ascertained at baseline or at the pre-COVID-19 assessment based on the data available in the PREDIMED-Plus database at the time of analysis. Physical activity was evaluated using the Regicor Short Physical Activity Questionnaire [21], and depressive symptoms using the Spanish version of the Beck Depression Inventory-II, which specifies a depressive symptomatology risk using a cut-off of >13 points[22, 23]. The adherence to the Mediterranean diet was assessed with the 17-point energy-restricted Mediterranean Adherence Screener (er-MEDAS), validated in the Spanish population [24].

For the main analysis, covariates were obtained from baseline or pre-COVID-19 visits. Covariates at baseline include sex (man; woman), recruitment center size (>400; 300-400; 250-300; <250 participants), educational level (<high school; high school; college), intervention group allocation, smoking status (never; former smoker; current smoker), and prevalence of type 2 diabetes (no; yes), hypertension (no; yes), and hyperlipidemia (no; yes). Covariates at the pre-COVID-19 assessment include participant’s age (years), marital status (single, divorced or separated; married; widower), body mass index (BMI; kg/m^2^), physical activity (METs min/week), alcohol intake adding the quadratic term (g/day), the respective cognitive function assessment (linear score), depressive symptomatology (no; yes), and having received at least one dose of COVID-19 vaccination (no; yes). Furthermore, the time elapsed between COVID-19 event status determination and the post-COVID-19 cognitive function assessment (in weeks) was included as a covariate.

#### Statistical analyses

This analysis used the PREDIMED-Plus database dated September 2023, merged with COVID-19 data. Missing covariates (<1%) were imputed as either the mean (continuous) or mode (categorical). Z-score of changes in cognitive domains were compared between COVID-19 positive and negative participants, adjusting for covariates. Linear and logistic regression models were used to assess associations between COVID-19 status (no/yes) and post-COVID-19 cognitive decline (post-COVID-19 z-scores) and impairment (absence/presence) for all cognitive domains. Crude, minimally adjusted, and three other models adjusting for potential covariates were tested. Supplementary analyses were performed for each cognitive test, using an alternative definition of cognitive impairment (1.5 SD below cohort pre-COVID-19 mean), and replacing pre-COVID-19 BMI, lifestyle and depression data with post-COVID-19 measurements.

**Table 2.** Comparison of changes of performance in various cognitive domains (z scores) from pre- to post-COVID-19 between COVID-19 cases and control (not infected) participants in the PREDIMED-Plus study.

Cognitive domain	COVID-19 Status						
	Positive			Negative			
	n	Change (95% CI)	p-value^1^	n	Change (95% CI)	p-value^1^	p-value^2^
**Global Cognitive function**	377	-0.09 (-0.15, -0.03)	<0.01	4,461	-0.18 (-0.19, -0.16)	<0.001	0.06
**General Cognitive function**	381	-0.15 (-0.27, -0.04)	<0.01	4,535	-0.30 (-0.33, -0.27)	<0.001	0.11
**Executive Function**	439	-0.08 (-0.14, -0.03)	<0.01	4,267	-0.10 (-0.11, -0.08)	<0.001	0.42
**Verbal Fluency**	445	-0.07 (-0.14, -0.00)	0.05	4,719	-0.07 (-0.09, -0.05)	<0.001	0.42
**Attention**	440	-0.04 (-0.04, 0.11)	0.36	4,680	-0.10 (-0.12, -0.07)	<0.001	0.23

Abbreviations: COVID-19, Coronavirus disease 2019; n, number of participants; 95% CI, 95% confidence interval. p-value1 calculated using paired t-test to assess differences of z-scores from pre-COVID-19 to post-COVID-19 assessments within groups. p-value2 calculated using ANCOVA to assess differences of z-score changes using delta method between COVID-19 status (negative/positive), adjusting for the respective cognitive domain performance at pre-COVID-19 (linear z-score), low and high Mediterranean diet adherence COVID-19 vaccine (no/yes), time elapsed between ascertainment of COVID-19 status and post-COVID-19 cognitive assessment (weeks), intervention group, center size (<250; 250-300, 300-400; ≥400 participants recruited), randomized as couples (no/yes), sex, smoking status (smoker; former smoker; never smoker),, educational level (primary school; secondary school; college), pre-COVID-19 covariates (age (in years), marital status (single, divorced or separated; married; widower), body mass index (kg/m2, and depressive symptomatology (no/yes)), physical activity (METs min/week), alcohol intake (g/day, adding the quadratic term), and prevalence of hypertension (no/yes), hypercholesterolemia (no/yes), type 2 diabetes (no/yes) at enrolment. The Global Cognitive Function domain was obtained by averaging all cognitive z-scores. The General Cognitive Function domain was obtained by averaging z-scores of the Mini-Mental State Examination and Clock Drawing Test. The Executive Function domain was obtained by averaging z-scores of the Verbal Fluency Test animals, Verbal Fluency Test letter “p”, Trail Making Test B, and the Digit Span Test backward. The Verbal Fluency domain was obtained by averaging z-scores of the Verbal Fluency Test animals and Verbal Fluency Test letter “p”. The Attention domain was obtained by averaging z-scores of the Trail Making Test A and the Digit Span Test forward.

## RESULTS

Of the 6,874 PREDIMED-Plus participants, 5,179 were included in this analysis inclusive of 448 COVID-19 positive cases (Supplementary Fig. 1). COVID-19-positive cases were more likely to be male, older, have higher education and BMI, and smoked less frequently compared to COVID-19-negative participants (Table 1).

Cognitive assessments post-COVID-19 in infected participants were performed at a mean (SD) of 50(29) weeks after infection, with only 13% assessed within 3 months of infection. Most (74%) post-COVID-19 assessments were performed 6 months post-infection.

Irrespective of COVID-19 status, all participants experienced reductions in scores of several cognitive domains from pre- to post-COVID-19 assessment, indicating cognitive decline. However, these changes did not differ by COVID-19 status when adjusted for potential covariates (Table 2, all p≥0.06).

No significant associations were observed between COVID-19 status and post-COVID-19 assessments for GenCF, Executive Function, Verbal Fluency, and Attention domains (Table 3, all p≥0.06). Accordingly, COVID-19 was not associated with post-COVID-19 cognitive impairment in these domains (Table 3, all p≥0.52). COVID-19 positive status was marginally associated with GCF measured post-COVID-19 (β_adj_ (95% CI): 0.06 (0.00, 0.13) p=0.05). However, cognitive impairment in GCF was not associated with COVID-19 (OR_adj_ (95% CI): 0.90 (0.53, 1.51) p=0.68).

No association between COVID-19 and cognitive decline or impairment was observed for any individual cognitive test (Supplementary Table S1). An alternative definition of cognitive impairment and the use of post-COVID-19 variables in supplementary analyses did not change the results (Supplementary Table 2).

**Table 3:** Association of COVID-19 with 50-week post-infection cognitive performance and impairment.

	Linear (β (95% CI))	p-value	Logistic (OR (95% CI))	p-value
**Global Cognitive Function (n=4,838)**				
**Model 1**	0.11 (-0.01, 0.22)	0.07	0.82 (0.57, 1.20)	0.31
**Model 2**	0.07 (0.01, 0.13)	0.03	0.87 (0.52, 1.45)	0.59
**Model 3**	0.06 (-0.00, 0.12)	0.05	0.91 (0.53, 1.54)	0.72
**Model 4**	0.06 (0.00, 0.13)	0.05	0.90 (0.53, 1.51)	0.68
**Model 5**	0.07 (0.01, 0.14)	0.02	0.87 (0.51, 1.48)	0.60
**General Cognitive Function (n = 4,916)**				
**Model 1**	0.10 (-0.01, 0.23)	0.08	0.86 (0.60, 1.24)	0.42
**Model 2**	0.11 (0.00, 0.22)	0.05	0.85 (0.57, 1.28)	0.43
**Model 3**	0.09 (-0.02, 0.20)	0.11	0.86 (0.57, 1.31)	0.50
**Model 4**	0.10 (-0.01, 0.23)	0.08	0.87 (0.58, 1.32)	0.52
**Model 5**	0.10 (-0.01, 0.23)	0.08	0.86 (0.57, 1.29)	0.46
**Executive Function (n= 5,066)**				
**Model 1**	0.07(-0.03,0.18)	0.16	0.84 (0.59, 1.19)	0.32
**Model 2**	0.02(-0.04, 0.08)	0.46	0.88 (0.56, 1.39)	0.59
**Model 3**	0.02(-0.04, 0.08)	0.46	0.91 (0.57, 1.43)	0.67
**Model 4**	0.02(-0.03, 0.08)	0.40	0.92 (0.58, 1.47)	0.74
**Model 5**	0.03(-0.03, 0.09)	0.37	0.90 (0.57, 1.43)	0.65
**Verbal Fluency (n = 5,164)**				
**Model 1**	0.07 (-0.02, 0.17)	0.14	1.02 (0.68, 1.53)	0.91
**Model 2**	0.03 ( -0.05, 0.10)	0.47	1.02 (0.68, 1.52)	0.94
**Model 3**	0.03 ( -0.05, 0.10)	0.47	1.02 (0.68, 1.54)	0.91
**Model 4**	0.03 ( -0.04, 0.10)	0.43	1.02 (0.68, 1.54)	0.90
**Model 5**	0.03 ( -0.04, 0.10)	0.41	1.00 (0.67, 1.50)	0.99
**Attention (n= 5,120)**				
**Model 1**	0.08 (-0.03, 0.19)	0.16	1.03 (0.74, 1.42)	0.88
**Model 2**	0.06 (-0.02, 0.14)	0.16	1.08 (0.72, 1.63)	0.71
**Model 3**	0.05 (-0.03, 0.13)	0.24	1.11 (0.73, 1.69)	0.62
**Model 4**	0.05 (-0.03, 0.13)	0.22	1.11 (0.73, 1.69)	0.62
**Model 5**	0.07 (-0.01, 0.15)	0.09	1.04 (0.68, 1.58)	0.86

Abbreviations: COVID-19, Coronavirus disease 2019; n, number of participants; β: beta coefficient from linear regression, OR: Odds ratio and 95% CI: 95% confidence interval. Linear regression models presented using β (95% CI) tested the associations between COVID-19 status (no/yes) and post-COVID-19 cognitive assessment (Z score: standardized change from pre-COVID-19 assessment). Logistic regression models using OR (95% CI) tested the associations between COVID-19 status (no/yes) and longitudinal post-COVID-19 cognitive impairment (absence/presence) in the 5 cognitive domains assessed using ≤10^th^ percentile performance for the cohort as a cut-off. Model 1: crude model. Model 2: adjusted by the respective cognitive domain performance at pre-COVID-19 (linear z-score), receipt of one dose of COVID-19 vaccine (no/yes), and time elapsed between COVID-19 status and post-COVID-19 cognitive assessment (in weeks). Model 3: Model 2 + additional adjustments for intervention group allocation, recruitment center size (<250; 250-300, 300-400; ≥400 participants), sex, educational level (primary school; secondary school; college) smoking status (smoker; former smoker; never smoker), pre-COVID-19 variables( (age (years), marital status (single, divorced or separated; married; widower), body mass index (kg/m^2^), adherence to Mediterranean Diet score ( on a 17-point scale), physical activity (METs min/week), and alcohol intake (g/day, adding the quadratic term)). Model 4: Model 3+ additional adjustments for participants’ disease prevalence at enrollment (diabetes, hypertension hypercholesterolemia) and elevated depressive symptomatology at pre-COVID-19 visit. Model 5: uses a minimal adjustment identified using a directed acyclic graph (DAG). This model includes sex, intervention group allocation, educational level (primary school; secondary school; college), smoking status (smoker; former smoker; never smoker), pre-COVID-19 variables (age (years), body mass index (kg/m^2^), adherence to Mediterranean Diet score (on a 17-point scale), physical activity (METs min/week), and alcohol intake (g/day, adding the quadratic term)), enrollment data on prevalence of diabetes( yes/no), prevalence of hypertension( yes/no), respective cognitive domain performance at pre-COVID-19 (linear z-score), and receipt of one dose of COVID-19 vaccine at pre-COVID-19 visit (no/yes).

## DISCUSSION

In a Spanish cohort of older adults with overweight/ obesity and MetS, we observed a small but significant cognitive decline between pre- and post-COVID-19 assessments, consistent with evaluations from the UK during the pandemic [25]. Nevertheless, contradicting several previous studies, we found that COVID-19 itself was not associated with cognitive decline or impairment post-infection, and even noted a small positive association for GCF with COVID-19 positive status, although clinically irrelevant. Earlier studies were limited in their ability to provide unbiased long-term estimates as they oftentimes lacked suitable controls or baseline cognitive scores, used hospitalized patients, self-reported cognitive measures, or measured cognition shortly after COVID-19 infection [4-7]. Our post-infection cognitive assessments were measured, on average, 1 year after diagnosis. Thus, the lack of association between COVID-19 and cognitive dysfunction documented here is likely to reflect the natural resolution with time [6]. It should also be noted that cognitive deficits were not detected in individuals who had reported a full recovery from COVID-19 [26]. Given that all participants included in this analysis were able to attend follow-up visits after COVID-19, it is probable that most cases included here were mild to moderate in severity and had a full recovery.

Our study aligns with recent recommendations for robust post-COVID-19 cognitive dysfunction evaluation [27]. We measured several cognitive domains using culturally validated methodology at pre- and post-COVID-19 visits, facilitating adjustment for individuals’ pre-COVID-19 data in both COVID-19-infected and similar controls without evidence of infection. The use of objective, validated, cognitive assessments is a particular strength of this study. It has been noted that a reliance on self-report instruments for the assessment of post-COVID-19 evaluation of cognitive dysfunction can produce skewed estimates due to the well-established dissonance between subjective and objective cognitive data. Additionally, utilizing the PREDIMED-Plus data allowed for accounting of recognized confounders of cognitive decline, and the analysis close to 1-year after infection provides a comprehensive perspective. Supplementary analyses including the use of post-COVID-19 covariates and alternative definitions of cognitive decline were consistent with the main findings, attesting to the robustness of our analysis. However, we acknowledge that we did not have data to account for COVID-19 severity, hospitalization, or infection strain which could determine post-COVID-19 sequalae [2, 4, 5]. Furthermore, we were unable to account for sleep duration and quality in the analysis, which may be associated with cognitive health, as we did not possess these data for the entire PREDIMED-Plus cohort. Therefore, residual confounding, while unlikely, cannot be discounted. The small number of COVID-19 cases in this cohort, despite reflecting the national prevalence at this time [28], could make this analysis underpowered to detect small but clinically significant differences in cognitive outcomes. It is also likely that misclassification of cases could have occurred as undiagnosed asymptomatic infections could have been labelled as COVID-19-negative. However, we also believe that this to have been highly unlikely as strategies for COVID-19 testing were stringent in Spain between 2020 and 2021, the period of COVID-19 ascertainment covered in this analysis. During this period, Spain was attempting to increase COVID-19 vaccination coverage and public health systems for COVID-19 testing were robust. Also, as described earlier, we included participants who attended both the pre-and post-COVID-19 cognitive assessments. Hence, it is highly likely that the analysis excluded those who developed severe complications. Thus, caution is required when extrapolating the results, as these findings may be generalizable only to those with mild and moderate COVID-19 who recovered without severe post-COVID-19 complications.

We recognize that our participants were enrolled in a lifestyle intervention program encouraging MedDiet in all participants and additional behavioral strategies for weight loss in the IG [10]. This could have attenuated any association of COVID-19 with cognition. It may also have been interesting to study structural brain changes through imaging studies to determine if changes in cognition parallel with changes in brain structure or function. However, current findings hold implications for public health preventive strategies, particularly in the context of documented detrimental lifestyle changes during COVID-19 restrictions [29] and campaigns to fight the spread of COVID-19 largely neglecting lifestyle improvement[29]. Considering the predicted increase in pandemics and the aging populations worldwide, strategies promoting healthy lifestyles could be crucial for public health.

In conclusion, among PREDIMED-Plus participants, there was no long-term association between COVID-19 status and cognitive decline or impairment. These findings must be interpreted in the context of a clinical trial that encouraged adherence to a healthy lifestyle, as the absence of evidence does not represent evidence of no effect.

## Supplementary Materials

The Supplementary data can be found online at: www.aginganddisease.org/EN/10.14336/AD.2024.0380.
