# Supplementary material for: COVID-19 and Cognitive Decline in Older Adults with High-Cardiovascular Risk: A Post Hoc Analysis
Source: Aging Dis. 2024 May 21;16(4):2373–82. doi: 10.14336/AD.2024.0380 (PMC12221412; doi:10.14336/AD.2024.0380)
Supplement: Supplementary file 1 — The Supplementary data can be found online at: www.aginganddisease.org/EN/10.14336/AD.2024.0380. [file AD-16-4-2373-s.pdf]

COVID-19 and Cognitive Decline in Older Adults with High-Cardiovascular Risk: A Post Hoc Analysis

Sangeetha Shyam, Carlos Gómez-Martínez, Jiaqi Ni, José J. Gaforio, Miguel Ángel Martínez-González, Dolores Corella, J. Alfredo Martínez, Ángel M. Alonso-Gómez MD, Julia Wärnberg, Jesús Vioque, Dora Romaguera, José López-Miranda, Ramon Estruch, Francisco J Tinahones, José Lapetra, Lluís Serra-Majem, Aurora Bueno-Cavanillas, Josep A. Tur, Vicente Martín-Sánchez, Xavier Pintó, Miguel Delgado-Rodríguez, Pilar Matía-Martín, Josep Vidal, Clotilde Vázquez, Lidia Daimiel, Emilio Ros, Fernando Fernandez-Aranda, Adrián Hernández-Cacho, Pilar Buil-Cosiales, Jose V. Sorli, Olga Castañer, Antonio Garcia-Rios, Alejandro Oncina-Canovas, Napoleón Pérez-Farinós, Mar Nafria, Rosa Casas, Silvia Martínez-Diz, Lucas Tojal-Sierra, Gómez-Pérez AM, Estefania Toledo, Rebeca Fernández-Carrión, Álvaro Morán Bayón, Jose David Torres-Peña, Laura Compañ-Gabucio, Zenaida Vázquez-Ruiz, Nancy Babio, Montserrat Fitó, Jordi Salas-Salvadó

Supplementary Table 1. Association of COVID-19 with post-COVID-19 cognitive decline or impairment in individual cognitive tests

Cognitive domain	n	Linear (β (95% CI))	p-value	Logistic (OR (95% CI))	*P*-value
MMSE	4,919	0.01 (-0.10, 0.11)	0.902	0.88 (0.61, 1.28)	0.501
CDT	4,919	0.11 (-0.02, 0.24)	0.090	0.74 (0.50, 1.09)	0.130
VFT-a	5,164	0.01 (-0.07, 0.09)	0.743	0.90 (0.64, 1.29)	0.577
VFT-p	5,164	0.04 (-0.03, 0.12)	0.279	0.88 (0.59, 1.33)	0.549
DST-f	5,151	0.03 (-0.06, 0.11)	0.560	1.10 (0.82, 1.47)	0.513
DST-b	5,151	-0.04 (-0.12, 0.04)	0.322	0.85 (0.60, 1.22)	0.378
TMT-A	5,131	-0.04 (-0.13, 0.04)	0.331	0.84 (0.57, 1.23)	0.370
TMT-b	5,075	-0.04 (-0.13, 0.03)	0.223	0.96 (0.65, 1.43)	0.847

Abbreviations: n, number of participants; OR (95% CI), β: beta coefficient from linear regression, OR: Odds ratio and 95% CI: 95% confidence interval; MMSE, Mini-Mental State Examination; CDT, Clock Drawing Test; VFT-a, Verbal Fluency Test animals; VFT-p, Verbal Fluency Test letter "p"; DST-f, Digit Span Test forward; DST-b, Digit Span Test backward; TMT-A, Trail Making Test A; TMT-B, Trail Making Test B.

Linear regression models presented using β(95% CI) tested the associations between COVID-19 status (no/yes) and post-COVID-19 cognitive assessment (Z score: standardized change from pre-COVID-19 assessment).

Logistic regression models, using OR (95%CI), were performed to assess the associations between COVID-19 status (no/yes) and longitudinal post-COVID-19 cognitive impairment (absence/presence) in all the cognitive tests assessed using $\leq 10^{th}$ percentile performance as cut-off

The model was adjusted by the respective cognitive domain performance at pre-COVID-19 (linear z-score), COVID-19 vaccine (no/yes), time elapsed between COVID-19 status and post-COVID-19 cognitive assessment (in weeks), intervention group, centre size (<250; 250-300, 300-400; ≥400), sex, smoking status (smoker; former smoker; never smoker), pre-COVID-19 covariates (age (in years), educational level (primary school; secondary school; college), marital status (single, divorced or separated; married; widower), body mass index (kg/m^2), physical activity (METs min/week), alcohol intake (g/day, adding the quadratic term) and depressive symptomatology (no/yes)), and prevalence of hypertension (no/yes), hypercholesterolemia (no/yes), type 2 diabetes (no/yes) at enrolment. Cluster variance estimator accounting for married couples was further employed.

SUPPLEMENTARY DATA

Supplementary Table 2. Association of COVID-19 with post-COVID-19 cognitive impairment.

	Logistic (OR (95% CI))	p-value
**Global Cognitive Function (n=4,838)**		
Crude Model	0.80 (0.57, 1.13)	0.206
Fully adjusted model	1.10 (0.66, 1.83)	0.711
Supplementary model with post-COVID-19 variables	0.80 (0.46,1.37)	0.414
**General Cognitive Function (n = 4,916)**		
Crude Model	0.90 (0.67, 1.20)	0.480
Fully adjusted model	1.07 (0.76, 1.51)	0.692
Supplementary model with post-COVID-19 variables	0.86 (0.56,1.32)	0.493
**Executive Function (n= 5,066)**		
Crude Model	0.83 (0.59, 1.17)	0.280
Fully adjusted model	0.90 (0.57, 1.42)	0.642
Supplementary model with post-COVID-19 variables	0.80 (0.48, 1.31)	0.368
**Verbal Fluency (n = 5,164)**		
Crude Model	0.96 (0.66, 1.41)	0.834
Fully adjusted model	1.16 (0.74, 1.84)	0.510
Supplementary model with post-COVID-19 variables	0.94 (0.61,1.44)	0.767
**Attention (n= 5,120)**		
Crude Model	0.86 (0.59, 1.28)	0.475
Fully adjusted model	0.93 (0.59, 1.47)	0.754
Supplementary model with post-COVID-19 variables	1.07 (0.70, 1.65)	0.753

Abbreviations: n, number of participants; β: beta coefficient from linear regression, OR: Odds ratio and 95% CI: 95% confidence interval.
Fully adjusted model for Logistic regression models, using OR (95%CI), tested the associations between COVID-19 status (no/yes) and longitudinal post-COVID-19 cognitive impairment (absence/presence) in the 5 cognitive domains assessed using ≤10th percentile performance for the cohort as a cut-off, adjusted by the respective cognitive domain performance at pre-COVID-19 (linear z-score), receipt of one dose of COVID-19 vaccine (no/yes), and time elapsed between COVID-19 status and post-COVID-19 cognitive assessment (in weeks), intervention group allocation, recruitment centre size (<250; 250-300, 300-400; ≥400), sex, age (years), educational level (primary school; secondary school; college), smoking status (smoker; former smoker; never smoker), pre-COVID-19 covariates (marital status at pre-COVID-19 visit (single, divorced or separated; married; widower), body mass index (kg/m^2), adherence to Mediterranean Diet score (on a 17-point scale), physical activity (METs min/week), and alcohol intake (g/day, adding the quadratic term)),participants' disease prevalence at enrollment (diabetes, hypertension and hypercholesterolemia).
Supplementary model with post COVID-19 variables for Logistic regression models, using OR (95%CI), tested the associations between COVID-19 status (no/yes) and longitudinal post-COVID-19 cognitive impairment (absence/presence) in the 5 cognitive domains assessed using -1.5SD from baseline performance for the cohort as a cut-off; adjusted by the respective cognitive domain performance at pre-COVID-19 (linear z-score), receipt of one dose of COVID-19 vaccine (no/yes), and time elapsed between COVID-19 status and post-COVID-19 cognitive assessment (in weeks), intervention group allocation, recruitment centre size (<250; 250-300, 300-400; ≥400), sex, age (years), educational level (primary school; secondary school; college), smoking status (smoker; former smoker; never smoker), marital status (single, divorced or separated; married; widower), post-COVID-19 covariates (body mass index (kg/m^2), adherence to Mediterranean Diet score (on a 17-point scale), physical activity (METs min/week), and alcohol intake (g/day, adding the quadratic term), depressive symptomatology (no/yes)), participants' disease prevalence (no/yes) at enrollment (diabetes, hypertension and hypercholesterolemia).

© 2024. Shyam S et al. Published online at http://www.aginganddisease.org/EN/10.14336/AD.2024.0380

SUPPLEMENTARY DATA

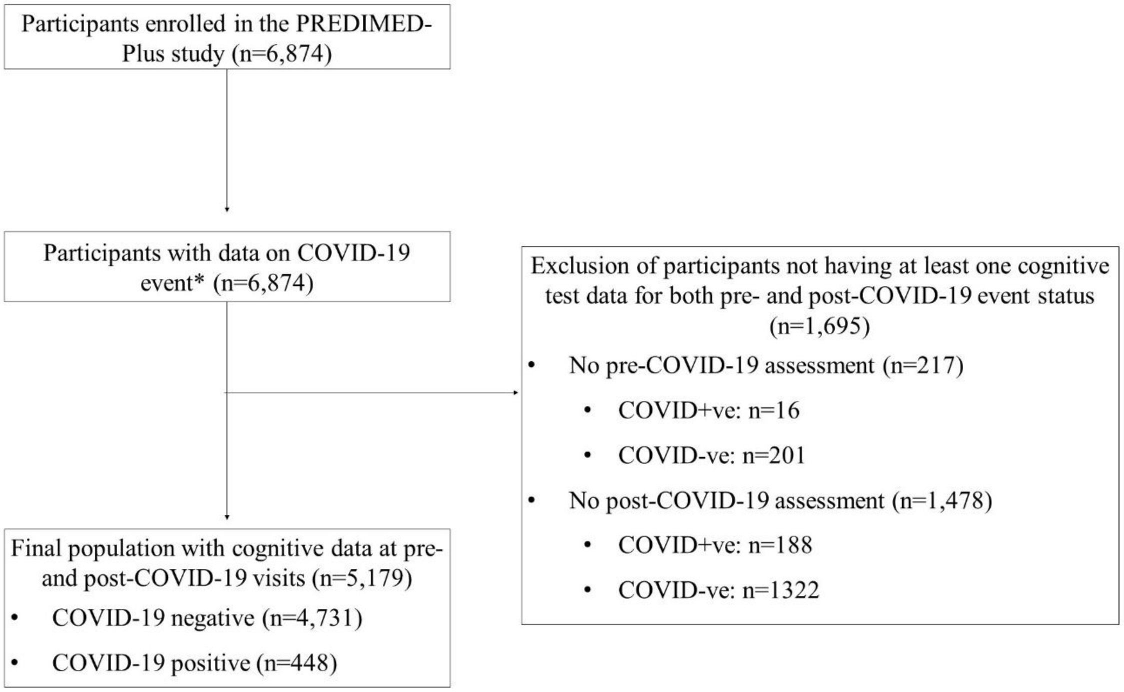

Supplementary Figure 1. Flowchart of the studied population. COVID-19 event status (yes/no) was ascertained as adjusted as by the event committee of the PREDIMED-Plus.

© 2024. Shyam S et al. Published online at http://www.aginganddisease.org/EN/10.14336/AD.2024.0380
